# Osteosarcoma Microenvironment: Whole-Slide Imaging and Optimized Antigen Detection Overcome Major Limitations in Immunohistochemical Quantification

**DOI:** 10.1371/journal.pone.0090727

**Published:** 2014-03-03

**Authors:** Pierre Kunz, Jörg Fellenberg, Linda Moskovszky, Zoltan Sápi, Tibor Krenacs, Johannes Poeschl, Burkhard Lehner, Miklos Szendrõi, Volker Ewerbeck, Ralf Kinscherf, Benedikt Fritzsching

**Affiliations:** 1 Department of Orthopedics and Traumatology, University Hospital Heidelberg, Heidelberg, Germany; 2 1st Department of Pathology and Experimental Cancer Research, Faculty of Medicine, Semmelweis University, Budapest, Hungary; 3 Department of Pediatrics, University Hospital Heidelberg, Heidelberg, Germany; 4 Department of Orthopedics, Semmelweis University, Budapest, Hungary; 5 Institute of Anatomy and Cell Biology, Department of Medical Cell Biology, University of Marburg, Marburg, Germany; 6 Department of Translational Pulmonology, Translational Lung Research Center (TLRC), Member of the German Center for Lung, University of Heidelberg, Heidelberg, Germany; University of Tübingen, Germany

## Abstract

**Background:**

In osteosarcoma survival rates could not be improved over the last 30 years. Novel biomarkers are warranted to allow risk stratification of patients for more individual treatment following initial diagnosis. Although previous studies of the tumor microenvironment have identified promising candidates, novel biomarkers have not been translated into routine histopathology. Substantial difficulties regarding immunohistochemical detection and quantification of antigens in decalcified and heterogeneous osteosarcoma might largely explain this translational short-coming. Furthermore, we hypothesized that conventional hot spot analysis is often not representative for the whole section when applied to heterogeneous tissues like osteosarcoma. We aimed to overcome these difficulties for major biomarkers of the immunovascular microenvironment.

**Methods:**

Immunohistochemistry was systematically optimized for cell surface (CD31, CD8) and intracellular antigens (FOXP3) including evaluation of 200 different antigen retrieval conditions. Distribution patterns of these antigens were analyzed in formalin-fixed and paraffin-embedded samples from 120 high-grade central osteosarcoma biopsies and computer-assisted whole-slide analysis was compared with conventional quantification methods including hot spot analysis.

**Results:**

More than 96% of osteosarcoma samples were positive for all antigens after optimization of immunohistochemistry. In contrast, standard immunohistochemistry retrieved false negative results in 35–65% of decalcified osteosarcoma specimens. Standard hot spot analysis was applicable for homogeneous distributed FOXP3^+^ and CD8^+^ cells. However, heterogeneous distribution of vascular CD31 did not allow reliable quantification with hot spot analysis in 85% of all samples. Computer-assisted whole-slide analysis of total CD31- immunoreactive area proved as the most appropriate quantification method.

**Conclusion:**

Standard staining and quantification procedures are not applicable in decalcified formalin-fixed and paraffin-embedded samples for major parameters of the immunovascular microenvironment in osteosarcoma. Whole-slide imaging and optimized antigen retrieval overcome these limitations.

## Introduction

Patients with osteosarcoma still suffer from a poor prognosis with a 5 year median survival of 30–75%. Since the introduction of neo-adjuvant chemotherapy thirty years ago therapeutic progress has been limited although most patients are enrolled in multi-center studies. Standard treatment protocols include uniform neo-adjuvant chemotherapy. Patients are not assigned to different treatment arms at the time of initial diagnosis after biopsy. Stratification is performed following preoperative chemotherapy and tumor resection based on histopathological assessment of the tumor response to chemotherapy. Currently presence of metastases is considered a key determinant of prognostic outcome together with response to chemotherapy, tumor size and site. However, metastases and chemotherapy response usually refer to later disease stages [1]. Improved risk stratification at the time of initial diagnosis is highly warranted but hampered by little progress in outcome-related histopathologic classifications and due to huge heterogeneity of osteosarcoma with numerous subentities [2]. Analysis of the tumor microenvironment might allow identification of parameters critical for prognosis independent of the osteosarcoma subtype and less affected by tumor heterogeneity. Furthermore, exploration of the immunovascular tumor microenvironment has been demonstrated to be of particular clinical value for prognosis and therapy of several other tumor entities [1], [3]–[9]. Although previous microenvironment studies have identified promising candidates [1], [10], [11], novel prognostic biomarkers have not been successfully translated into standard osteosarcoma histopathology. Major technical difficulties like non-standardized immunohistochemistry protocols and varying quantification methods are currently limiting translation of novel biomarkers into clinical routine [12]–[18]. In decalcified, morphological heterogeneous specimens like osteosarcoma, these difficulties are of exceptional relevance [19]–[21]. In contrast to emerging quantification techniques [8], [22]–[27], the current gold standard for quantification of tumor microenvironment relies on conventional immunohistochemical analysis of subjectively selected hot spots [8], [17], [28]. However, it is even unclear if such hot spot areas can be defined in all tissues analyzed since hot spot based quantification originates from histopathological examination of carcinomas with homogeneous morphologies and abundant hot spot distribution patterns of vessels and tumor infiltrating cells [8], [17], [28], [29]. Application of hot spot analysis on tumor entities with vast heterogeneity like osteosarcoma retrieves conflicting results [30]–[34]. We hypothesize that hot spots might often not be representative for the whole section with heterogeneous distribution patterns of tumor vessels and tumor infiltrating lymphocytes. Correct quantification is further hampered by inconsistent detection of numerous antigens [19], [20], [34], [35]. No consensus quantification algorithm for immunovascular parameters in osteosarcoma specimen is currently available.

Therefore, we evaluated 120 decalcified, formalin-fixed and paraffin-embedded high-grade central osteosarcoma samples from two European centers. We considered CD31, CD8 (cell surface epitopes) and FOXP3 (intracellular epitope) as immunovascular biomarkers of major interest in osteosarcoma, representing intratumoral vessels (CD31), tumor attacking T-cells (CD8) and immunosuppressive or anergic T-cells (FOXP3). After optimization and standardization of epitope retrieval protocols we analyzed the distribution patterns of these biomarkers and compared hot spot and computer-assisted whole-slide quantification methods for immunovascular parameters to establish a reliable and feasible quantification algorithm for immunohistochemical analysis of osteosarcoma biopsies.

## Materials and Methods

### Patient specimens

All formalin-fixed and paraffin-embedded specimens of open high grade central osteosarcoma biopsies were randomly selected from the University of Heidelberg and the University of Budapest bone tumor bank.

### Ethics statement

Ethical approval was obtained by the ethical committee of the faculty of medicine, University of Heidelberg. General sampling of tumor biopsies was approved by the ethics committee vote 207/2005. This study in specific was approved by the ethics committee vote 312/2006. The study was conducted in accordance to the declaration of Helsinki.

### Immunohistochemistry

Most samples were acid-decalcified by either 10–20% formic acid (68% of samples), 10% EDTA (17,5%) or other agents (14%). Standard immunohistochemistry protocols for CD31, FOXP3 and CD8 were used as a starting point for systematic optimization of antigen retrieval, antigen detection by primary antibodies and visualization by different labeling systems and chromogens as shown in table 1 [26], [29], [36].

**Table 1:** Parameters included in systematic evaluation of staining conditions for CD31, CD8 and FOXP3 in osteosarcoma samples.

**Retrieval**	DRS pH 6, 98°C, 30 min
	EDTA pH 7–9, 98°C, 30 min
	Citrat ph 6, 98°C, 30 min
	DRS pH 6, 110°C–136°C*, 5–15 min**
	EDTA pH 7–9, 110°C–136°C*, 5–15 min**
	Citrat pH 6, 110–136°C*, 5–15 min**
	Chymotrypsin, Proteinase 24, Proteinase K, Pronase, Hyaluronidase ***
**Blocking**	No blocking
	BSA, 5%/10%
	Human Serum, 5%/10%
**Antibodies**	CD31 (JC70A)
	FOXP3 (236A/E7)
	CD8 (C8/144B)
**Detection systems**	Vectastain ABC-AP Kit (Vector Labs)
	Vectastain Elite ABC-HRP Kit (Vector Labs)
	Ultravision LP detection system HRP (Polymer, Thermo Fisher)
	Ultravision LP detection system AP (Polymer, Thermo Fisher)
	EnVision+HRP labelled Polymer (Dako)
**Chromogens**	Liquid Fast Red (Thermo Fisher)
	Fast Red (Roche)
	3,3′-Diaminobenzidine (DAB) (Vector Labs)
	3,3′-Diaminobenzidine (DAB) + Nickel (Vector Labs)
	3-amino-9-ethylcarbazole (AEC) (Vector Labs)
	Histogreen (Linaris)

Systematic evaluation of more than 200 different antigen retrieval conditions, different blocking conditions, detection systems and chromogens allowed identification of optimal staining conditions for formalin-fixed and paraffin-embedded osteosarcoma samples.

* In steps of 3–5°C;

** in steps of 5 min;

*** in concentrations of 0.1%–10%, adjusted temperatures and various times and combinations.

Best results were retrieved as follows: After deparaffinization and rehydration of sections antigen retrieval for CD31 detection was performed by incubation with 5% Pronase (30 min, 37°C), followed by three times washing in PBS and incubation with 0,1% Hyaluronidase (30 min, 37°C). FOXP3 and CD8 antigen retrieval was performed in a CertoClav EL pressure cooker (Certoclav, Traun, Austria) at 127° for 15 min in EDTA buffer with pH 7,0 for FOXP3 or pH 8,0 for CD8.

After three times washing in PBS and blocking with 10% human serum in PBS, osteosarcoma cross sections were covered by cover plates and transferred to slide racks (Thermo Shandon Limited, Astmoor, UK). CD31 mAb (clone JC70A, Dako, Glostrup, Denmark) was diluted 1∶30 in 10% human serum and incubated (2 h, room temperature). FOXP3 mAb (clone236A/E7, eBioscience, San Diego, USA) and CD8 mAb (clone C8/144B, Dako) were diluted 1∶50 in 10% human serum and incubated (16 h, 4°C).

Alkaline phosphatase labeling was performed by polymer linked secondary antibody (UltraVision LP, Thermo Fisher Scientific, Waltham, USA) and fast red tablets (Roche, Rotkreuz, Suisse) for CD31. For Alkaline phosphatase based detection of FOXP3 and CD8 liquid fast red (Thermo Fisher Scientific, Waltham, USA) was used. After staining (25 min), sections were washed in PBS, and counterstained with hematoxylin (1 min).

### Digital Imaging and quantification

Immunohistochemical stained osteosarcoma sections were digitalized using a Scanscope CS slide scanner from Aperio (Vista, USA) and tumor areas were differentially annotated by an experienced bone pathologist using the Aperio ImageScope software. Quantification of vascularization was performed by computer assisted determination of CD31- immunoreactive area either for the whole section or within three annotated vascular hot spots (0,26 mm^2^ each) using the Aperio micro vessel algorithm. The mean analyzed tumor area was 70 mm^2^ representing approximately 300 hot spots. Slides with an evaluable area below 5 mm^2^ were excluded. 90% of all included slides showed an analyzed area of vital, non- perinecrotic tumor area of at least 40 mm^2^. To minimize effects of heterogeneous staining intensities color threshold settings were optimized for each osteosarcoma cross section. Chalkley and micro vessel density counts were performed manually within the annotated hot spots as previously described by others [8], [28], but adapted for digitalized slides. In brief, a 25-dot Chalkley graticule was applied to hot spots and was adjusted to allow a maximum number of dots to touch or lay within stained vessels. These dots were counted. Micro vessel density was determined by counting singular identifiable vessels within annotated hot spots.

For quantification of tumor infiltrating lymphocytes digital color deconvolution was performed and immunoreactive cells were manually counted within the area of the whole section or the area of hot spots. All analyses were conducted by two independent investigators. Interobserver variability was determined as follows: mean values of the analyzed cohort were calculated and interobserver difference values for a given slide were compared to mean values. Interobserver variability was considered acceptable when below 10%.

### Statistical analysis

Correlations of different vessel quantification methods (micro vessel density, Chalkley count and total stained area) and correlations of whole-slide and hot spot analyses for tumor vascularization and tumor infiltrating lymphocytes were determined by the Pearson correlation coefficient. Statistical tests are based on two-sided testing with the significance defined as p<0.05. Kaplan-Maier curves were used for estimating the survival function from lifetime data. All statistics were performed using SPSS software (IBM, Armonk, NY).

## Results

### Enzymatic CD31 antigen retrieval is superior to heat-induced antigen retrieval in decalcified osteosarcoma samples

**Figure 1:**
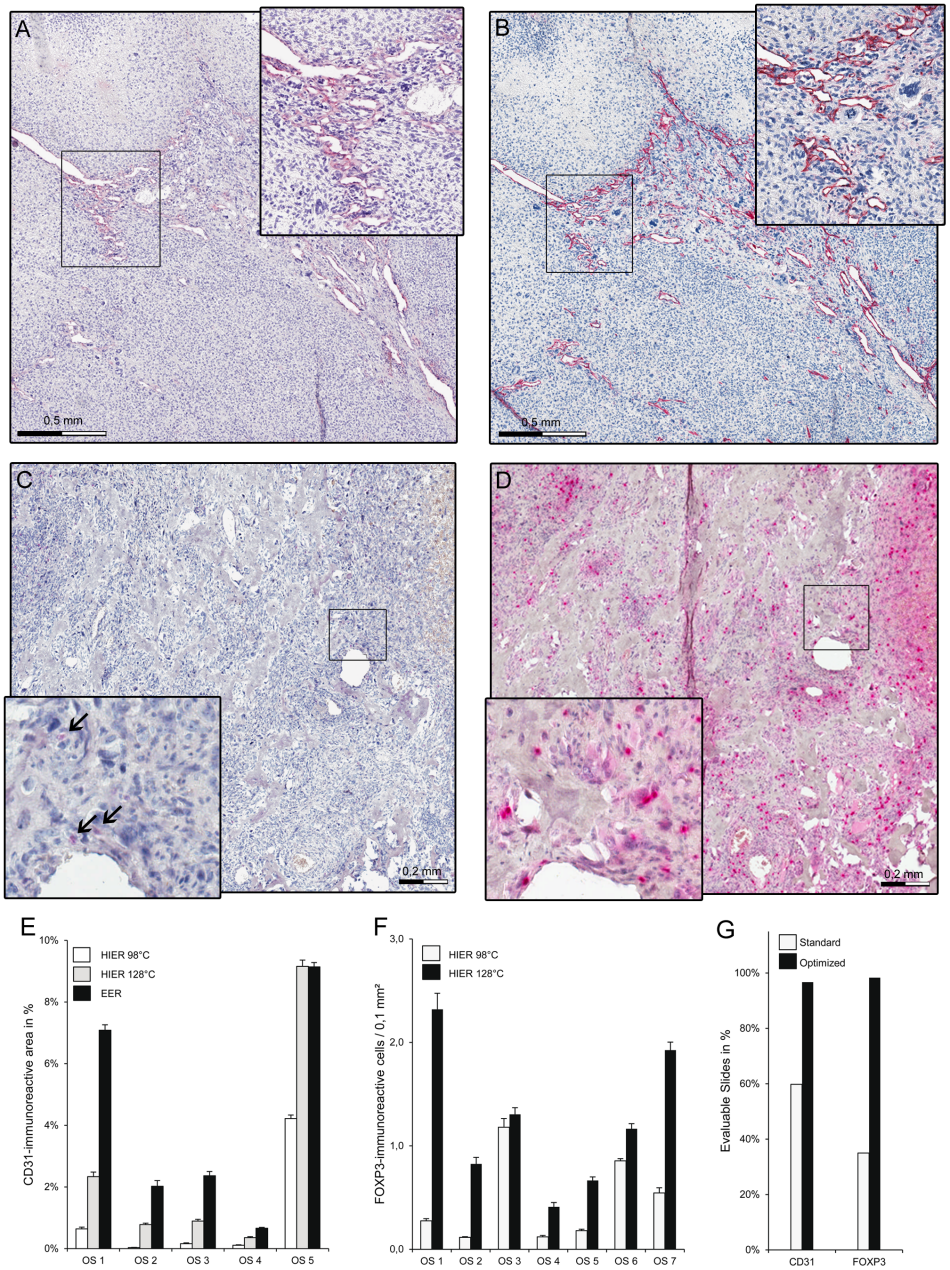
Effects of different staining conditions on the quantification of immunovascular markers in osteosarcoma. (A) Formalin-fixed and paraffin-embedded osteosarcoma sample after CD31 staining with standard heat induced epitope retrieval at 98°C and (B) with optimized enzymatic epitope retrieval. CD31- immunoreactive cells show red cell surface staining. Section was counterstained by hematoxylin. Insert shows 2-fold magnification of indicated area. (C) Formalin-fixed and paraffin-embedded osteosarcoma sample after FOXP3 staining with standard heat induced epitope retrieval at 98°C and (D) with optimized epitope retrieval at 127°C. FOXP3 immunoreactive cells (arrows) show red nuclear staining. Section was counterstained with hematoxylin. Insert shows 3.8 fold magnification of indicated area. (E) Percentage of CD31 immunoreactive area was assessed by computer-assisted whole-slide quantification after heat induced epitope retrieval (HIER) at 98°C, HIER at 127° and enzymatic epitope retrieval (EER) with Hyaluronidase and Pronase in five representative osteosarcoma samples (OS). Error bars indicate interobserver variability. (F) Only seven out of the 20 tested osteosarcoma samples showed FOXP3 immunoreactive cells after standard heat induced epitope retrieval at 98°C (not shown). Density of FOXP3-immunoreactive cells (numbers/0.1 mm^2^) was determined in these seven sections by whole-slide quantification after HIER at 98°C and for HIER at 127°C. Error bars indicates interobserver variability. OS = osteosarcoma sample. (G) Percentage of evaluable slides after standard and optimized immunohistochemical staining for CD31 and FOXP3.

When we applied recommended heat-induced epitope retrieval protocols for detection of CD31 [30], [31], [37] in decalcified, formalin-fixed and paraffin-embedded osteosarcoma samples, results were false negative in approximately 35% of all analyzed samples (n = 120). 51% of analyzed samples seemed evaluable, but most samples showed false low staining results. In addition, we observed high variability in staining intensities. Systematic evaluation of different conditions for antigen retrieval (Table 1) in serial sections revealed that enzymatic antigen retrieval in combination with polymer detection is superior to heat-induced antigen retrieval and conventional avidin-biotin complex based detection (Fig. 1A,B). Even heat-induced antigen retrieval with temperatures above 117°C for at least 15 min was less efficient than optimized enzymatic antigen retrieval (Fig. 1E). Optimized staining conditions allowed an evaluation of more than 96% of all analyzed samples (Fig. 1G). Of note, even under optimized conditions, staining intensities still varied somewhat throughout different specimens, reflecting the heterogeneous antigenicity in osteosarcoma specimens (Fig. 1E). We did not see relevant differences in CD31 immunoreactivity depending on the decalcification solution used. Furthermore, no signs of inadequate decalcification like altered hematoxylin staining were observed.

### High pressure heat-induced epitope retrieval allows reliable detection of nuclear FOXP3 and cell surface CD8 antigens in decalcified osteosarcoma samples

We and others have previously established an optimized protocol for FOXP3 immunohistochemistry using heat-induced epitope retrieval at 98°C in EDTA pH 9 for 30 min in various tissues [29], [38]–[40]. However, when we applied this protocol on osteosarcoma specimens, we retrieved inconsistent staining intensities with false low detection of FOXP3 positive cells in 35%, and negative detection of FOXP3 positive cells in 65% of all tested samples (n = 20) (Fig. 1C,F). Systematic evaluation of different antigen retrieval protocols (Table 1) revealed high pressure heat-induced epitope retrieval in combination with polymer detection as a reliable method for detection of CD8 and FOXP3 in decalcified osteosarcoma sections. In fact, under these conditions FOXP3^+^ cells could be detected in 98% of 120 analyzed osteosarcoma samples which could further be evaluated (Fig. 1G).

Furthermore, in contrast to standard detection methods, polymer detection counteracted an increased background staining frequently observed by reactivated endogenous biotin during the harsh retrieval process for FOXP3 and CD31 antigen.

### Whole-slide analysis but not hot spot analysis allows correct quantification of the vascular microenvironment of osteosarcoma specimens

**Figure 2:**
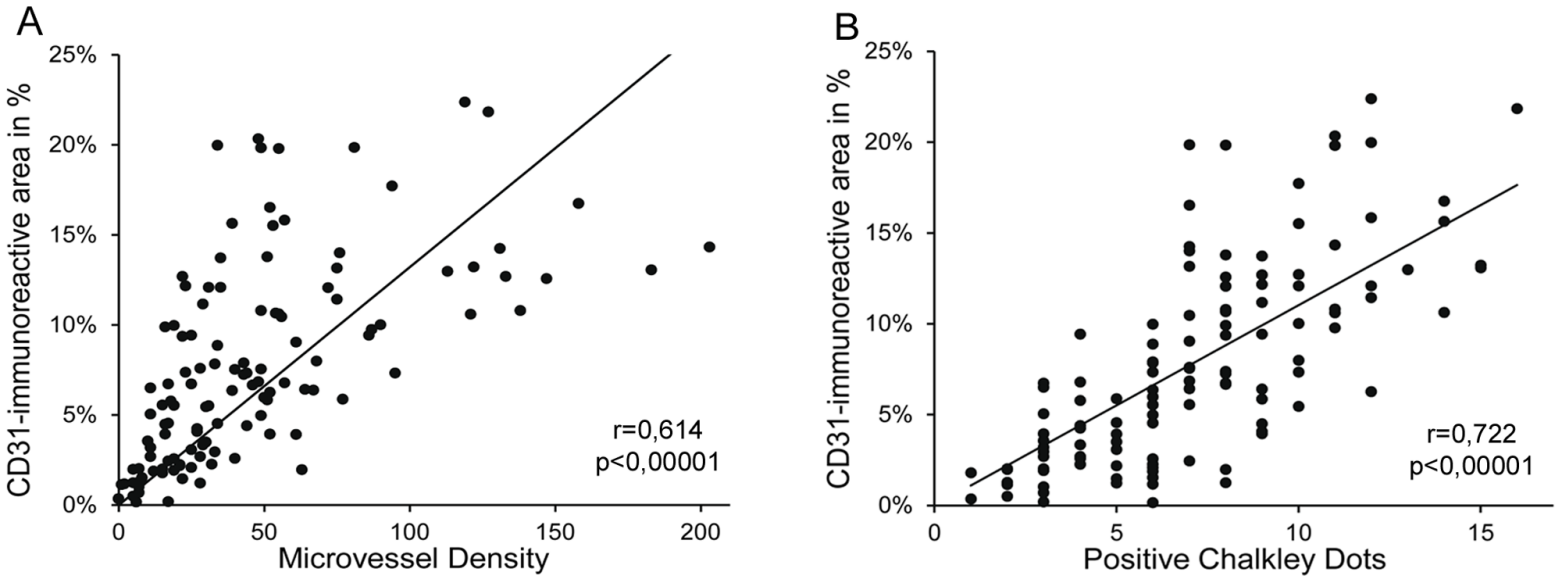
Correlation of common vessel quantification methods in osteosarcoma. Correlation of vessel quantification derived by total CD31-immunoreactive area and micro vessel density (A), respectively total CD31- immunoreactive area and Chalkley count (B) within 120 predefined spots with 0.26 mm^2^ area/spot in 20 representative osteosarcoma sample; r indicates Pearson correlation coefficient.

**Figure 3:**
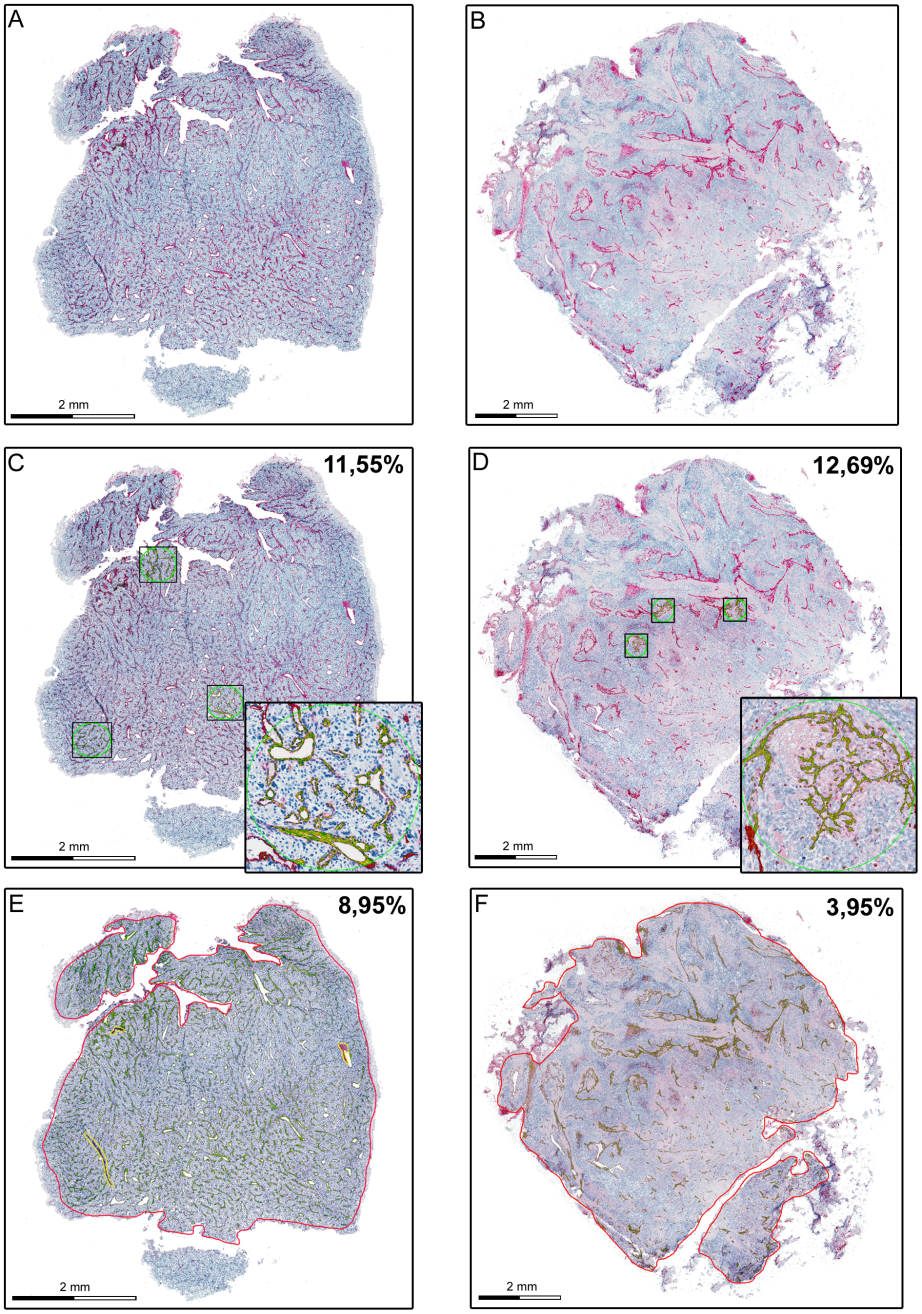
Effect of heterogeneous vessel distribution on vessel quantification in osteosarcoma. Representative whole-slide scans of formalin-fixed and paraffin-embedded osteosarcoma samples with homogeneously scattered (A,C,E) and hot spot distributed intratumor vascularization (B,D,F) Quantification of tumor vascularization was either performed by hot spot analysis within three circular hot spots with 0.26 mm^2^ area/hot spot (C and D) or whole-slide analysis of CD31-immunoreactive area (E and F). CD31 immunoreactivity is shown in red. By digital image analysis detected CD31-immunoreactive area is annotated in green (automated mark-up image). Indicated values represent the percentage of immunoreactive area within the analyzed regions (three hot spots in C and D, whole slide in E and F). Inserts show a 5-fold (C), respectively 8-fold (D) magnification of indicated regions. Sections were counterstained by hematoxylin.

**Figure 4:**
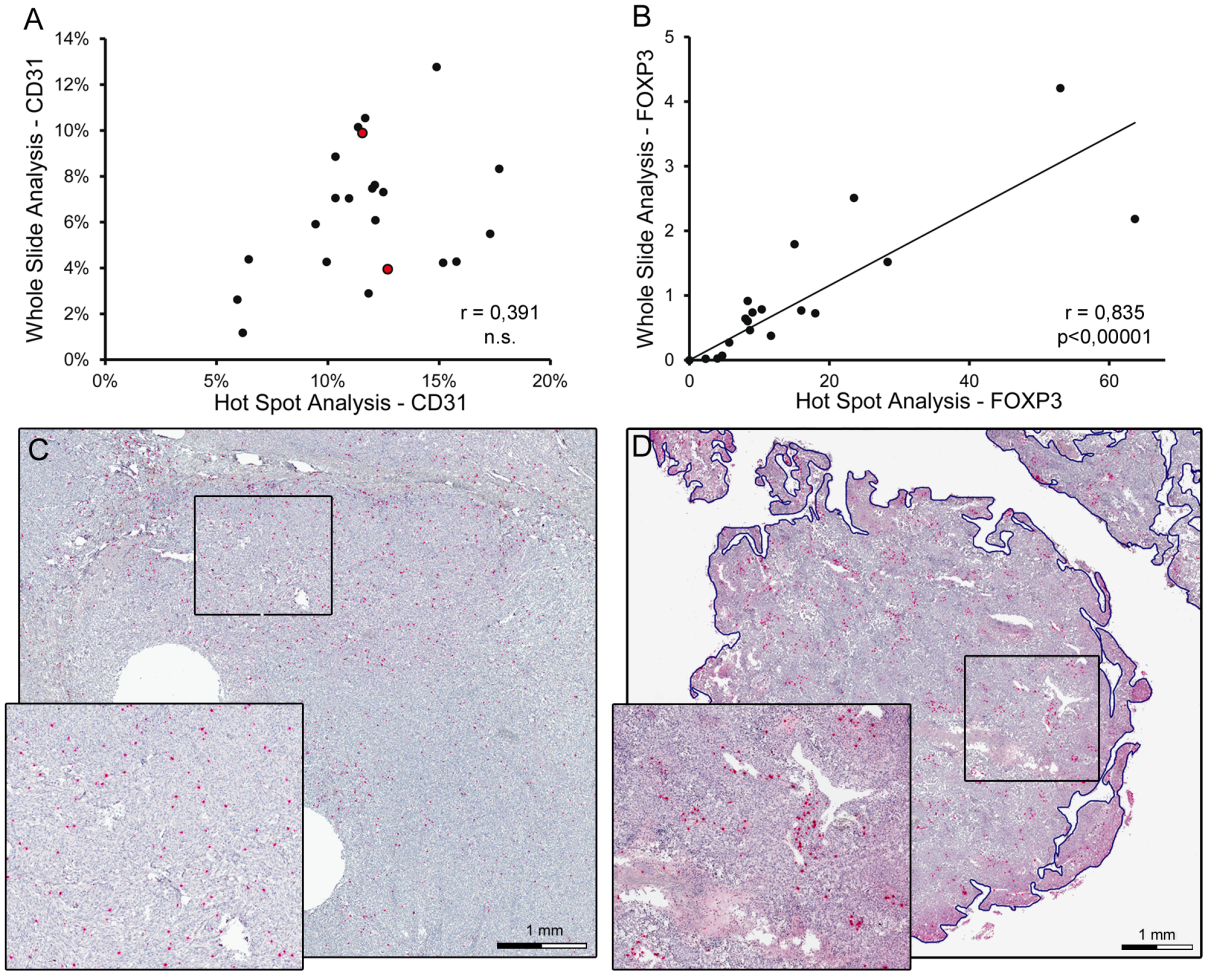
Correlation of hot spot analysis and whole slide analysis of immunovascular markers in osteosarcoma. (A) Correlation between hot spot and whole slide analyses of CD31-immunoreactive area (in %) of 20 representative osteosarcoma samples. Circles filled in red represent the two specimens shown in Figure 3. Pearson correlation coefficient is indicated by r, n.s. =  not significant. (B) Correlation between hot spot and whole slide analyses of FOXP3 cell density (cells per 0.1 mm^2^) of 20 representative osteosarcoma samples. Pearson correlation coefficient is indicated by r and significance by p. (C) Representative osteosarcoma sample with homogeneously scattered distribution of FOXP3 immunoreactive cells. Immunoreactive cells show red nuclear staining. Section was counterstained by hematoxylin. Insert shows 2.2 fold magnification of indicated area. (D) Representative osteosarcoma sample with homogeneous hot spot distribution of CD8 immunoreactive cells. Immunoreactive cells show red cell surface staining. Section was counterstained by hematoxylin. Insert shows 2.2 fold magnification of indicated area.

Quantification of marker expression in hot spots can be performed either by determination of total stained area, micro vessel density or Chalkley counting [8], [28], [41]. When we compared our results obtained by the quantification of the CD31- immunoreactive endothelial area with micro vessel density or Chalkley counting in 120 spot areas we found a strong and highly significant correlation in both cases (Fig. 2A,B). These data confirm CD31- immunoreactive endothelial area as a good correlate for micro vessel density or Chalkley counts in preselected spots. Next, we tested if hot spot analysis of CD31-immunoreactive area correlates with vessel quantification of the whole section. To this end, whole sections were scanned and digital image data processing was applied (Fig. 3A,B). In 20 representative osteosarcoma samples CD31-immunoreactive area ranged from 1,17% to 12,77% with a median of 6, 57%±2, 89% (SEM). Complete section size covered an average of 0.7 cm^2^, whereas standard hot spot analysis includes three hot spots covering less than 1.1% of the whole section. When we compared whole-slide analysis with hot spot analysis we could not observe any significant correlation (r = 0.391, Fig. 4A). Careful side by side reanalysis revealed high heterogeneity of the immunovascular microenvironment as the interfering factor. Samples with homogeneous vessel distribution of CD31 displayed a comparable amount of CD31- immunoreactive area in the whole section and in hot spots. (Fig. 3C,E). In contrast, samples with heterogeneous distribution of CD31 showed a much higher percentage of CD31- immunoreactive area in hot spots than in the whole section (Fig. 3D,F). Application of standard hot spot analysis was further limited by heterogeneously scattered vascularization allowing evident hot spot definitions only in 15% of 120 evaluated osteosarcoma specimens. In addition, we compared interobserver variability in hot spot and whole-slide analysis. Due to the absence of clear-cut vascular hot spots in 85% of osteosarcoma specimens, interobserver congruency of selected hot spots was achieved in only 40% of all hot spots. In contrast, whole-slide analysis remained unaffected by this issue and showed the lowest interobserver variability (data not shown).

### Homogeneous distributed tumor infiltrating lymphocytes allow both hot spot and whole-slide analysis for correct quantification in osteosarcoma specimens

In 20 representative osteosarcoma samples whole-slide numbers of FOXP3^+^ cells ranged from 0,003 to 4,1 cells/0,1 mm^2^ with a median of 0,73 cells/0,1 mm^2^±1,06 (SEM). Semiquantitative whole-slide assessment of CD8^+^ cells revealed a high CD8^+^ cell frequency in 30%, an intermediate CD8^+^ cell frequency in 25%, and a low CD8^+^ cell frequency in 45% of osteosarcoma samples. In contrast to the vascular microenvironment, we observed significant correlation of FOXP3^+^ cell counts derived by whole-slide analysis and hot spot analysis (r = 0,835; p<0,00001, Fig. 4B). Reassessment of the immunostained osteosarcoma cross sections revealed a homogeneous distribution of FOXP3 immunoreactive cells (Fig. 4C) in 70% of 120 samples which might explain this correlation. Similarly, CD8^+^ cells were distributed homogeneously in 80% of analyzed osteosarcoma samples (n = 120) (Fig. 4D). We conclude that hot spot analysis might be applicable for homogenous distributed but not heterogeneous distributed elements of the osteosarcoma microenvironment.

## Discussion

In our effort to establish a reliable algorithm to quantify important parameters of the immunovascular microenvironment in osteosarcoma, we focused on optimization of antigen retrieval and antigen quantification in formalin-fixed and paraffin-embedded samples from osteosarcoma biopsies. Two major difficulties of osteosarcoma specimens need to be considered in particular: tissue decalcification and tumor heterogeneity. Analysis of the tumor microenvironment might not only allow identification of parameters critical for osteosarcoma prognosis. The presented algorithm might also be applicable to other tumor entities with a similar heterogeneous distribution of microenvironmental antigens.

When we applied recommended staining protocols for osteosarcoma specimens [26], [29]–[31], [37], we could verify that the applied methods for antigen retrieval and detection greatly influence the reliability of immunohistochemical quantification in osteosarcoma. Systematic evaluation of staining procedures for major immunovascular biomarkers (CD31, FOXP3 and CD8) revealed that standard staining protocols did not allow consistent antigen detection. Conventional detection of CD31^+^ micro vessels was false negative in 35% out of 120 high grade central osteosarcoma specimens, and was false negative for FOXP3 positive cells in 65% out of a representative group of 20 osteosarcoma samples. Moreover, false low staining for CD31 and FOXP3 was detected in the vast majority of analyzed osteosarcoma samples. Conflicting results in previous studies of osteosarcoma vascularization [30]–[34], [37] and the lack of publications demonstrating FOXP3 positive T_reg_ in human osteosarcoma might arise from this issue. Different decalcification conditions including formic acid decalcification or EDTA did not affect antigen detection (data not shown).

The presented protocols established during our study for cell surface antigens (CD31, CD8) and intracellular/nuclear antigens like FOXP3 might be also used for other antigens in formalin-fixed and paraffin-embedded osteosarcoma samples and probably other tumors.

However, we cannot rule out that detection of other antigens and application of other immunostaining protocols might depend on specific decalcification conditions. Recommended decalcification solutions still vary depending on the specific sample. Whereas bone tumor specimens are reported to frequently require 10% formic acid or even stronger acids [42], small biopsies with little mineralized bone fragments or bone-marrow trephines are suggested to be decalcified in EDTA [43]. Given an ongoing controversy on antigen stability during decalcification, standardized decalcification in osteosarcoma immunohistochemistry is warranted.

From an idealistic view, a biopsy should be representative for a tumor and immunohistochemical work up should allow quantitative detection of all detectable antigens and should include the whole tissue specimen. In this regard, there is an ongoing discussion about representativeness of a biopsy in heterogeneous tumors like osteosarcoma and correct quantification strategies of immunostained samples. At first, it still remains unknown for the surgeon before taking a biopsy which area of a heterogeneous tumor hosts the most responsible cells for clinical outcome. Even, if the surgeon would know this area he has to consider the future resection approach when taking the biopsy and must avoid tumor cell spreading, i.e. due to hematoma. Given these limitations, also open biopsies remain a compromise between biopsy representativeness and success of the subsequent limb salvage tumor resection. Similarly, current quantification methods are limited by under- or overestimation of correct antigen numbers and by limitations of time-consuming quantification strategies [17]. Conventional immunohistochemistry can only derive estimated values for true antigen density and distribution from a given tumor specimen. Correlations of such estimated values with clinical outcome are frequently performed to gain novel predictors for clinical course and prognosis. As a requirement for this approach, it is assumed that the quantified area represents the whole specimen. However, in heterogeneous tumor samples such representative areas need to be much larger for reliable analysis compared to homogeneous tumors. Further requirements for correct correlations include high reproducibility, low interobserver variability, traceability of the obtained results and time and cost efficiency for a possible routine application [17], [44].

Conventional quantification of vascularization includes different methods like hot spot analysis of micro vessel density, vessel lumen, perimeter, total stained area or whole-slide analysis, which have all been demonstrated to allow more or less reproducible vessel quantification [8], [17], [23], [45], [46]. Eligibility for above mentioned requirements strongly depends on the investigated tissue. The majority of available data on tumor vascularization emerged from carcinoma tumor entities [8], [17], [41], [46] with homogeneous hot spot distribution of vessels and moderate intervessel variability [17], [47]. In homogeneous carcinomas quantification of 3–5 selected vascular hot spots for micro vessel density or Chalkley counts allows consistent correlations with clinical outcome in a time and cost efficient fashion and is therefore considered as the gold standard for intra tumor vessel quantification in carcinomas [8], [17], [28]. However, limited applicability of hot spot analysis in tumors without homogenous vessel distributions is well known and has been highlighted for sarcomas in current consensus reports on vessel quantification [17], [47]. In our effort to characterize the immunovascular microenvironment of 120 osteosarcoma specimens, we confirmed the limitation of hot spot derived quantification in such heterogeneous tissue. No significant correlation between whole-slide analysis and hot spot analysis was found in a representative cohort of 20 osteosarcomas from the total group of 120 osteosarcoma samples. When we reanalyzed sections with similar vascularization values as determined by hot spot analysis, whole-slide analysis revealed broad heterogeneity of vascularization and retrieved dissimilar results (data not shown). We therefore concluded that selected hot spots were not representative for the whole section in osteosarcoma. In general, hot spot analysis might still be considered useful for clinically relevant characteristics of the entire tumor: The original idea of hot spot analysis in tumor vascularization was based on the assumption that the vascular density within vascular hot spots represents tumor clones with the highest angiogenic capacity and therefore determines the probability of haematogenic metastasis and survival [17]. However, three major issues need to be considered. First, increasing numbers of hot spots with high angiogenic capacity within a given tumor area could increase the chance of metastasis. Second, hot spot regions with high vascularization are in fact much more variable in size than standardized hot spot areas, angiogenic capacity might be further misjudged from hot spot analysis. Third, high numbers of less intense vascularized areas within heterogeneous sections could outweigh reduced vascularization in hot spots and therefore alter the risk of metastasis. In contrast to hot spot analysis, these issues are considered in whole-slide analysis.

With the broad availability of computer-assisted antigen quantification, whole-slide analysis might overcome the short-comings of hot spots analysis. However selection of a vessel quantification method requires careful attention:

Quantification of micro vessel density, vessel lumen or perimeter depends on a reliable identification of single vessels despite interruptions of endothelial lining by tissue tears or lack of staining. Careful adjustment of various software settings is mandatory and software settings cannot easily be adapted from one specimen to another specimen. Furthermore, verification by the investigator is highly time-consuming.

CD31-immunoreactive area appears to be a more robust and as demonstrated above equivalent parameter. Adjustment of software settings is limited to staining intensity threshold for each slide since singular vessels have not to be identified. Results can easily be verified by the investigator on automatically generated mark-up images (see Fig 3C–F).

Furthermore, preliminary data supports the relevance of optimized CD31 detection and whole-slide imaging for their use as a potentially useful surrogate parameter for clinical outcome. In a representative group of 20 osteosarcoma specimens, Kaplan-Maier estimated survival was determined for samples with low, intermediate or high CD31- immunoreactive area (Fig. S1). Although the small cohort size of 20 patients and variations in clinical follow-up periods certainly narrow the validity of statistical interpretation, high CD31- immunoreactivity could be recognized as a significantly worse outcome parameter in osteosarcoma patients (p = 0,031). To test these preliminary findings, a large study cohort of more than 120 osteosarcoma samples will be analyzed (unpublished data). In a pilot study, combined anti-angiogenic therapy with endostatin and multidrug chemotherapy showed potential to prevent the progression of metastases [48]. Given the heterogeneity of the vascular microenvironment, subgrouping of osteosarcoma patients along above mentioned CD31 data might serve as a biomarker to identify patients with further benefit including increased survival.

In summary, whole-slide analysis of total immunoreactive area but not hot spot analysis fulfills most requirements for reliable quantification of heterogeneously distributed antigens like CD31 in osteosarcoma samples. In this line, we observed the lowest interobserver variability in whole-slide analysis compared to other quantification methods like hot spot analysis (data not shown).

In general, above discussed issues apply to all elements of the immunovascular microenvironment in osteosarcoma. However, in case of more homogeneous distributed antigens like FOXP3, quantification by whole-slide analysis and hot spot analysis provide comparable results, as shown by a significant correlation in a representative cohort of 20 osteosarcoma specimens.

In this regard it should be considered that comparison of two or more parameters is frequently required to reveal interdependencies within the immunovascular microenvironment. Comparison of hot spot data for one parameter with whole section data for the other parameter should be avoided.

In case one parameter is distributed heterogeneously (i.e. CD31) whereas the other is distributed rather homogenously (i.e. FOXP3), whole-slide analysis should be preferred for both parameters.

## Conclusion

In our effort to establish a reliable quantification algorithm for important immunovascular antigens in formalin-fixed and paraffin-embedded osteosarcoma biopsies, we consider whole-slide analysis and standardized antigen retrieval as indispensable elements to overcome limitations due to decalcification and heterogeneity of the tumor microenvironment in osteosarcoma. Furthermore we defined optimal staining procedures for detection of major immunovascular cell populations and their standardized quantification by whole-slide analysis. The presented algorithm might also serve for immunohistochemical analysis of other heterogeneous tumor entities.

## Supporting Information

Figure S1 **Kaplan-Maier estimated survival of osteosarcoma patients with low, intermediate or high CD31- immunoreactive area in pretreatment biopsies.** Kaplan-Maier estimated survival of 20 osteosarcoma patients grouped for CD31- immunoreactive area in pretreatment biopsies. Upper dashed line: patients with low CD31- immunoreactive area (n = 5). Dotted line: Patients with intermediate CD31- immunoreactive area (n = 9). Lower dashed line: Patients with high CD31- immunoreactive area (n = 6). Overall survival indicated in months. Cumulative survival indicated in percent.
(TIF)
