# Supplementary figures and images for: Osteosarcoma Microenvironment: Whole-Slide Imaging and Optimized Antigen Detection Overcome Major Limitations in Immunohistochemical Quantification
Source: PLoS One. 2014 Mar 3;9(3):e90727. doi: 10.1371/journal.pone.0090727 (PMC3940945; doi:10.1371/journal.pone.0090727)

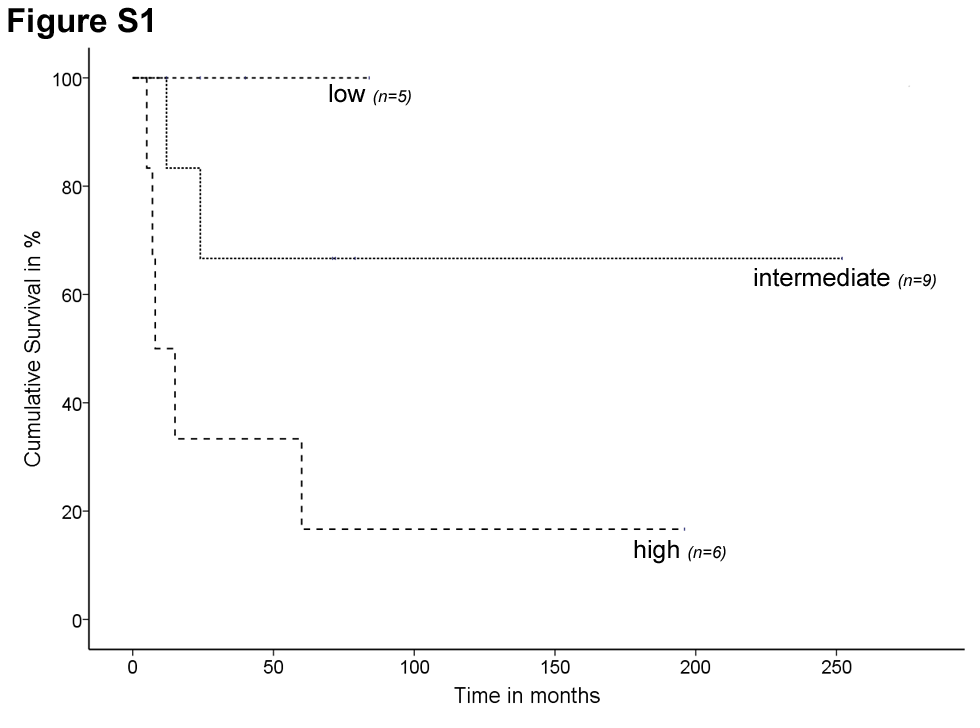

Supplement: Figure S1 — Kaplan-Maier estimated survival of osteosarcoma patients with low, intermediate or high CD31- immunoreactive area in pretreatment biopsies. Kaplan-Maier estimated survival of 20 osteosarcoma patients grouped for CD31- immunoreactive area in pretreatment biopsies. Upper dashed line: patients with low CD31- immunoreactive area (n = 5). Dotted line: Patients with intermediate CD31- immunoreactive area (n = 9). Lower dashed line: Patients with high CD31- immunoreactive area (n = 6). Overall survival indicated in months. Cumulative survival indicated in percent. (TIF) [file pone.0090727.s001.tif]
